# Endogenous theta stimulation during meditation predicts reduced opioid dosing following treatment with Mindfulness-Oriented Recovery Enhancement

**DOI:** 10.1038/s41386-020-00831-4

**Published:** 2020-09-12

**Authors:** Justin Hudak, Adam W. Hanley, William R. Marchand, Yoshio Nakamura, Brandon Yabko, Eric L. Garland

**Affiliations:** 1 Center on Mindfulness and Integrative Health Intervention Development, University of Utah, Salt Lake City, UT USA; 2 College of Social Work, University of Utah, Salt Lake City, UT USA; 3 Veterans Health Care Administration VISN 19 Whole Health Flagship site located at the VA Salt Lake City Health Care System, 500 Foothill, Salt Lake City, UT 84148 USA; 4 Department of Psychiatry, University of Utah School of Medicine, 501 Chipeta Way, Salt Lake City, UT 84108 USA; 5 Department of Anesthesiology, Division of Pain Medicine, Pain Research Center, University of Utah School of Medicine, Salt Lake City, UT 84108 USA

**Keywords:** Human behaviour, Addiction

## Abstract

Veterans experience chronic pain at greater rates than the rest of society and are more likely to receive long-term opioid therapy (LTOT), which, at high doses, is theorized to induce maladaptive neuroplastic changes that attenuate self-regulatory capacity and exacerbate opioid dose escalation. Mindfulness meditation has been shown to modulate frontal midline theta (FMT) and alpha oscillations that are linked with marked alterations in self-referential processing. These adaptive neural oscillatory changes may promote reduced opioid use and remediate the neural dysfunction occasioned by LTOT. In this study, we used electroencephalography (EEG) to assess the effects of a mindfulness-based, cognitive training intervention for opioid misuse, Mindfulness-Oriented Recovery Enhancement (MORE), on alpha and theta power and FMT coherence during meditation. We then examined whether these neural effects were associated with reduced opioid dosing and changes in self-referential processing. Before and after 8 weeks of MORE or a supportive psychotherapy control, veterans receiving LTOT (*N* = 62) practiced mindfulness meditation while EEG was recorded. Participants treated with MORE demonstrated significantly increased alpha and theta power (with larger theta power effect sizes) as well as increased FMT coherence relative to those in the control condition—neural changes that were associated with altered self-referential processing. Crucially, MORE significantly reduced opioid dose over time, and this dose reduction was partially statistically mediated by changes in frontal theta power. Study results suggest that mindfulness meditation practice may produce endogenous theta stimulation in the prefrontal cortex, thereby enhancing inhibitory control over opioid dose escalation behaviors.

## Introduction

Veterans are more likely to experience pain than the general population [1], with 29% of Veterans reporting chronic pain [2]. Between 2010 and 2016, 25% of Veterans with chronic pain received an opioid prescription [1, 3]. Among its risks, long-term opioid therapy (LTOT) at high doses is theorized to induce maladaptive neuroplasticity in prefrontal, limbic, and striatal circuitry, reducing self-regulatory capacity and promoting opioid dose escalation as a means of preserving hedonic equilibrium [4, 5]. To mitigate opioid-related harms, the Department of Veterans Affairs (VA) launched Opioid Safety Initiatives to decrease opioid prescribing and improve safety monitoring. In response to these initiatives, use of complementary and integrative therapies like mindfulness-based interventions (MBIs) has significantly increased across the VA healthcare system [3].

MBIs provide training in focused attention and open monitoring meditation techniques [6]. Focused attention is a self-regulatory practice that involves sustained attention on a target (most often body sensations like the breath), executive attention to notice and prevent mind wandering, and reorienting of attention back to the intended target [7]. Open monitoring builds on the attentional stability cultivated during focused attention. During open monitoring, the practitioner relaxes attentional focus on the target, adopting a form of ambient attention and meta-awareness that monitors the arising and ceasing of mental contents from moment-to-moment while reflecting upon the “background” of awareness in which those mental contents arise. As open monitoring reaches its zenith, practitioners often report marked alterations in self-referential processing in the form of ego dissolution, a softening of perceived body boundaries, and a blissful, phenomenological unity between self and world [8, 9]. Though such “nondual” self-transcendent states are often assumed to be inaccessible to only but the most advanced practitioners, recent psychometric research indicates that they occur even among novices [10–12].

Electroencephalography (EEG) has been used to study the neural mechanisms of meditation [13, 14]. Meta-analyses of EEG spectral activity occasioned by mindfulness meditation identified frontal midline alpha and theta synchronization as the primary EEG biomarkers of mindfulness [15, 16]. Alpha (9–13 Hz) activity, traditionally thought to represent states of hypoarousal [17], increases in prefrontal regions as the practitioner enters the meditative state [18]. Later, both frontal alpha and theta (4–8 Hz) power increase as the practitioner sustains and deepens the meditative state [18]. During this latter phase of meditation, it is thought that alpha band synchronization reprises its classical role by maintaining low cortical arousal despite perturbations by external stimuli [13], while theta band synchronization subserves recruitment of meta-awareness and cognitive control to sustain the meditative state [19]. In addition to spectral power increases, meditation has also been shown to increase coherence (i.e., connectivity) across distal neural populations, wherein simultaneously measured EEG channels synchronize across time within a particular spectral band. In particular, frontal midline theta (FMT) coherence has been observed in multiple studies of mindfulness and other meditation practices [16, 18, 20–23]. FMT coherence and power are thought to reflect increased prefrontal (PFC) and anterior cingulate cortex (ACC) activation during maintenance and deepening of the meditative state [18, 23].

**Table 1:** Baseline demographic and clinical characteristics (*N* = 62) of Veterans treated with Mindfulness-Oriented Recovery Enhancement (MORE) or a supportive group (SG) psychotherapy control condition.

Measure	MORE	SG	Test statistic	*p*-value
*N* (%)	34 (55%)	28 (45%)		
Female, *N* (%)	3 (11%)	6 (18%)	*χ*^2^(1, *N* = 61) = 0.58	0.45
Age, M ± SD	60.2 ± 9.8	58.1 ± 10.3	*t*(60) = −0.79	0.43
Race, *N* (%)^a^			*χ*^2^(4, *N* = 61) = 0.36	0.99
African American	1 (3%)	1 (4%)		
Hispanic/Latino	2 (6%)	1 (4%)		
White	28 (82%)	23(82%)		
Native American/American Indian	2 (6%)	1 (4%)		
Other	1 (3%)	1 (4%)		
Primary pain location, *N* (%)^a^			*χ*^2^(4, *N* = 61) = 3.50	0.48
Back	19 (55%)	15 (56%)		
Legs feet	2 (6%)	2 (7%)		
Joints	6 (18%)	3 (11%)		
Neck/Shoulders	3 (9%)	6 (22%)		
Other	4 (12%)	1 (4%)		
Opioid type^b^			*χ*^2^(5, *N* = 61) = 1.97	0.85
Oxycodone	10 (29%)	9 (33%)		
Hydrocodone	8 (24%)	9 (33%)		
Tramadol	13 (38%)	7 (26%)		
Morphine	3 (9%)	3 (11%)		
Methadone	3 (9%)	1 (4%)		
Other	4 (12%)	3 (11%)		
Opioid use duration (years)	9.4 ± 7.1	8.7 ± 9.2	*t*(60) = −0.33	0.74
Average Pain, M ± SD	5.4 ± 1.4	5.4 ± 1.5	*t*(60) = −0.09	0.93
Morphine equivalent daily dose, M ± SD	94.6 ± 207.9	98.4 ± 216.6	*t*(60) = 0.07	0.95

^a^Subject demographic and pain location data were missing for 1 subject in the SG.

^b^Subjects were allowed to enter more than 1 opioid type.

The overwhelming majority of EEG studies of mindfulness meditation have been conducted in non-clinical populations. To address this lacuna in the literature, we sought to examine mindfulness-induced changes in EEG spectral activity among a sample of Veterans on LTOT participating in Mindfulness-Oriented Recovery Enhancement (MORE), a MBI designed to target the comorbidity of chronic pain and opioid misuse. In two Stage 2 randomized controlled trials (RCTs), relative to a supportive psychotherapy control, MORE significantly decreased opioid misuse and pain severity among people receiving LTOT [24, 25], therapeutic effects that were associated with mindfulness-induced self-transcendence. In addition, MORE decreased opioid dosing by 32% and the opioid sparing effects of MORE were mediated by increases in heart rate variability during mindfulness mediation [26].

Here, in an ancillary mechanistic sub-study overlaid on a clinical trial (NCT02935621), we used EEG to test whether participation in MORE versus supportive group (SG) psychotherapy would occasion increased alpha and theta power as well as FMT coherence during a laboratory-based mindfulness meditation session. Further, we hypothesized that increased theta power would be associated with altered self-referential processing during the meditative state and predictive of decreased opioid dosing following treatment with MORE.

## Methods

### Participants

This study evaluated EEG data from a sample of Veterans receiving LTOT (*N* = 62). Primary clinical outcomes from NCT02935621 will be reported elsewhere. Individuals with complete pre-post treatment EEG data (MORE, *n* = 34; SG, *n* = 28) were included in the present analysis (Table 1). Participants (85% male; mean age = 59.3 ± 9.9) were recruited from VA primary care and pain clinics, and met inclusion criteria if they reported chronic non-cancer pain (mean pain duration = 16.4 ± 12.9 years) and had taken opioids for at least the past 90 days (mean opioid use duration = 9.1 ± 8.1 years). The mean (±SE) opioid dose in morphine milligram equivalents (MME: 96.3 ± 26.68 mg) fell within the “high dose” range (>90 MME) as specified by the Centers for Disease Control [27]. With regard to co-occurring non-opioid substance use, on the day of the pre-treatment EEG session, four participants tested positive for benzodiazepines and eight participants tested positive for THC on urine toxicology screening. Participants were excluded if they had previously engaged in a formal MBI or for active suicidality or psychosis as assessed by the Mini-International Neuropsychiatric Interview [28]. Participants were financially compensated. The protocol was approved by the University of Utah IRB and VA Salt Lake City Health Care System Research and Development Committee, and all procedures complied with standards set forth in the Helsinki Declaration of 1975.

### Procedures

Following screening, participants who gave informed consent completed demographic and clinical assessments. Next, participants were interviewed by a clinically-trained research assistant using the Timeline Followback [29] to assess daily opioid use. Then they completed a laboratory-based, mindfulness practice session during which EEG was recorded. Participants were informed that they would be randomized to a behavioral treatment group that would help them to cope with pain, stress, and opioid-related problems by providing either mindfulness training or supportive group psychotherapy. After the pre-treatment assessment, participants were randomly allocated to MORE or a SG control. The allocation sequence was generated via computerized random number table by a researcher who was uninvolved in assessment, treatment, or enrollment using simple randomization in blocks of varying sizes (2–4) to preserve allocation unpredictability. Assessments were conducted by research staff blinded to group assignment (which remained concealed throughout the study). After participants completed the 8-week MORE or SG treatment, they returned to the lab to complete a post-treatment assessment consisting of the Timeline Followback and the laboratory-based, mindfulness practice session with EEG recordings. Participants returned for 2- and 4-month follow-ups where they again completed the Timeline Followback.

### Interventions

The manualized MORE intervention program provided training in mindfulness, reappraisal, and savoring skills as techniques to cope with opioid craving, pain, and negative affect [30]. Group sessions were 2-h long and led by a psychologist. Mindfulness training involved mindful breathing and body scan techniques to help patients self-regulate pain and opioid craving, with additional meditation instructions to induce a nondual state of consciousness marked by decreased self-referential processing and a fading of body boundaries [11, 12]. Participants were asked to engage in daily 15-min mindfulness sessions at home guided by an audio recording. In addition, participants were asked to pause before taking their next opioid dose and practice three minutes of mindful breathing—a practice intended to disrupt habitual (automatic) use of opioid and increase self-awareness of whether opioid use was driven by craving or a need for pain relief. Ultimately, these techniques were intended to strengthen self-regulatory capacity and reduce unneeded opioid dosing.

To control for non-specific factors, including attention by a caring professional, therapeutic expectancy, and social support, we employed a manualized active SG control in this study. The SG consisted of 8 weekly, 2-h Rogerian group psychotherapy sessions, in which a psychologist facilitated emotional expression and discussion of topics pertinent to chronic pain and opioid use/misuse. This client-centered SG format was validated in two RCTs of MORE [24, 25]. SG participants were asked to engage at home in 15 min of journaling a day on chronic pain and opioid-related themes. To prevent treatment diffusion, participants in the SG condition were instructed to not engage in mindfulness training during the course of the study. A clinician with 15+ years of experience conducted clinical supervision and reviewed session recordings to monitor therapist adherence to the MORE and SG treatment manuals and maintain intervention fidelity.

### Measures

#### EEG during laboratory-based mindfulness meditation practice

EEG was continuously recorded from 10 midline scalp sites (Fz, F3, F4, FC1, FC2, FCz, Cz, CP1, CP2, and PZ) using an active sensor cap with Ag/AgCl electrodes (actiCap GmbH, Herrsching, Germany). Additionally, vertical electro-oculograms (EOG) were recorded. All recordings were collected by an actiCHamp amplifier (Brain Products GmbH, Gilching, Germany). Data were acquired at a sampling rate of 500 Hz, a resolution of 0.489 μV and an amplification cutoff of 140 Hz, with impedances kept below 10 kΩ. EEG was recorded during a 10-min mindfulness meditation practice. All participants received the same instruction: “Now practice mindfulness, which means focusing on your thoughts, feelings and body sensations in the present moment in a nonjudgmental way, without reacting to them.” In keeping with methods used in previous mindfulness studies [26, 31], to control for demand characteristics, these task instructions were kept constant across both treatment conditions (MORE and SG), allowing us to isolate the effects of mindfulness training through the MORE intervention from any potential instruction effects. We assumed that meditation-naïve participants randomized to the SG control would be unable to successfully practice mindfulness meditation with such nondescript instructions, whereas participants randomized to MORE would be able to successfully employ these simple instructions to cue them to practice the combined FA and OM mindfulness technique taught during the 8-week MORE intervention. The meditation was comprised of two 5-min blocks (eyes open and eyes closed); for this study, EEG spectral frequency power and coherence values were averaged across the 5-min eyes closed portion of the meditation at the pre- and post-treatment assessments.

#### Opioid dose

Average daily opioid dose throughout the study was assessed using the validated Timeline Followback [29]. Self-reports of opioid dosing data were triangulated through electronic health record review of opioid prescription data. Opioid dose was converted to MME using standardized equianalgesic conversions [27].

#### Mindfulness-induced changes in self-referential processing

As a manipulation check, we examined whether treatment with MORE increased self-reported changes in self-referential processing during the laboratory-based mindfulness meditation session, as measured by the Nondual Awareness Dimensional Assessment (NADA-state; [10]) and the Perceived Body Boundaries Scale (PBBS; [32]. The NADA-state is a 3-item scale that assesses ego dissolution (e.g., sense of the self dissolving or the experience of oneness) and associated blissful sensations during meditation using an 11-point Likert Scale (0 = not at all, 10 = very much). The PBBS is a single item visual scale that assesses the strength of the experiential boundary between self and world using a 7-point Likert scale (1 = weak boundary, 7 = strong boundary).

### EEG Data reduction

All EEG analyses were performed in MATLAB [33] using scripts implementing the EEGLAB toolbox [34]. In a first step, epochs of 300 s were created for each subject, corresponding to the eyes closed meditation. A notch filter at 60 Hz was then applied to account for line noise. Then, the signal was low-pass filtered at 40 Hz cutoff using a butterworth filter of the 4th order and high-pass filtered at 0.1 Hz to using a butterworth filter of 2nd order, to account for noise not within expected EEG frequency spectra. Filtered data were subsequently passed through the PREP pipeline using the default parameters. Briefly, the PREP pipeline [35] detrends the data, applies a notch filter tapering off the harmonics of 60 Hz, re-references the data to the linked-earlobe, and identifies and interpolates bad channels (<10% of data were interpolated). Interpolation in low-density montages has been shown to produce valid data [35–38]. In power spectral density analyses, average power spectra for both the theta (4–8 Hz) and alpha (9–13 Hz) bands were calculated using Welch’s periodogram. These averages were then averaged again over ROIs specified in prior mindfulness studies [20, 21]: frontal (F3, F4, Fz), frontal midline (FCz, FC1, FC2, Cz), and parietal (Pz, CP1, CP2). For the spectral coherence (i.e., functional connectivity) analyses, the squared coherence magnitude was calculated between each channel and within the respective frequency bands, normalized using a Fisher’s Z transform and finally averaged over the aforementioned ROIs. Separate sensitivity analyses of the EEG data were conducted after removing artifacts identified by visual inspection, and this did not alter the significance or directionality of the hypothesized treatment effects.

### Statistical analysis

This mechanistic study was powered to detect changes in EEG spectra. Given that MORE produced a moderate sized effect on heart rate variability during meditation (*ƞ*_partial_^2^ = 0.07) in civilians receiving LTOT [26], we assumed a moderate effect size of MORE on EEG spectral power. Assuming a Cohen’s *f* = 0.25 for the Group X Time interaction and a repeated measures correlation *r* = 0.30, power = 0.90 with *N* = 62.

For hypothesis testing, first we conducted repeated-measures ANOVAs (RM-ANOVAs) to test whether MORE led to greater increases in spectral power in alpha and theta bands relative to the SG. RM-ANOVA models included a time factor (pre- vs. post-treatment), an ROI factor, a between-subjects treatment factor (MORE vs. SG), and a treatment X time X ROI interaction. A similar RM-ANOVA tested the whether MORE increased theta coherence relative to the SG (i.e., *FMT hypothesis*).

Next, to assess the effects of treatment on opioid dose over time, we used a linear mixed modeling approach with maximum likelihood estimation and fixed effects consisting of a time factor and between-subjects treatment factor (MORE vs. SG). The parameter of interest was the treatment X time interaction, and the model was specified with a random intercept. This mixed model was adjusted for pre-randomization differences in opioid dose by covarying pre-treatment MME.

To test whether increases in spectral power mediated the effect of treatment on reduced opioid dose, we conducted a path analysis with maximum likelihood estimation using AMOS 24.0 software to evaluate pre-post changes in spectral power as a mediator of treatment effects (MORE vs. SG) on changes in opioid dose from pre-treatment to 4-month follow-up, with mediation indicated by a significant Sobel test. The Sobel test is conservative and resistant to outliers in small samples [39], and has a lower Type I error rate than asymptotic boostrapping approaches under the same power scenario [40]. Recent Monte Carlo simulations demonstrate that a sample size of 50–60 is required to detect a significant indirect effect with the Sobel test when both the ‘a’ and ‘b’ paths are of moderate effect sizes [41].

Finally, to test whether increases in spectral power during meditation were associated with changes in self-referential processing from pre- to post-treatment, we computed regression models in AMOS 24.0 in which change in spectral power served as the predictor of NADA and PBBS scores at post-treatment, controlling for pre-treatment levels. We also computed Pearson correlations to examine associations between mindfulness meditation practice duration (number of minutes a day) and spectral power; one outlier was removed for high practice duration (≥3 SD).

## Results

### Effects of MORE on spectral power during meditation

**Fig. 1:**
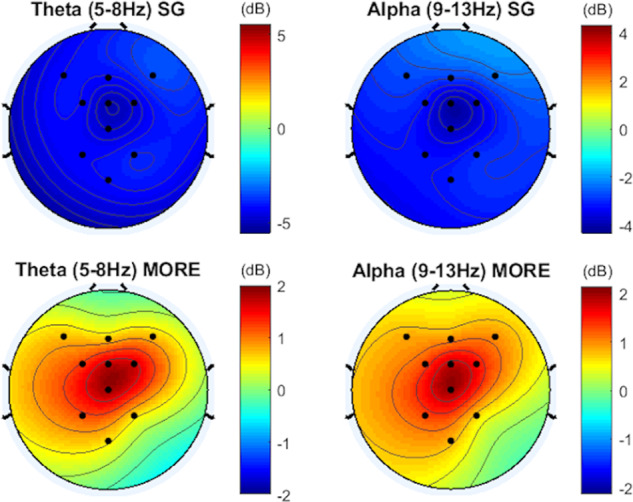
Spectral EEG changes during a laboratory-based mindfulness meditation practice session before and after 8 weeks of mindfulness-oriented recovery enhancement (MORE) or a supportive group (SG) psychotherapy control condition. Topoplots are interpolated to cover the entire headspace. dB = decibels.

The RM-ANOVA for theta power changes revealed a treatment x time interaction, *F*(1,60) = 7.33, *p* = 0.009 (Fig. 1). Planned post-hoc comparisons of between-groups difference scores at each ROI revealed moderate to large effect sizes for increases in frontal (*d* = 0.65), frontal midline (*d* = 0.68), and parietal (*d* = 0.70) theta power in the MORE group relative to SG. Similarly, alpha power changes also showed a treatment x time interaction *F*(1,60) = 6.26, *p* = 0.02. Planned post-hoc comparisons of between-groups difference scores at each ROI revealed moderate to large effect sizes for increases in frontal (*d* = 0.57), frontal midline (*d* = 0.60), and parietal (*d* = 0.67) alpha power in the MORE group relative to SG. No treatment X time X ROI was observed, indicating that alpha and theta increased in the MORE group across all ROIs. In a sensitivity analysis controlling for opioid dose, as well as recent benzodiazepine and THC use, treatment X time interactions for theta and alpha power remained statistically significant.

### Effects of MORE on theta coherence during meditation

**Fig. 2:**
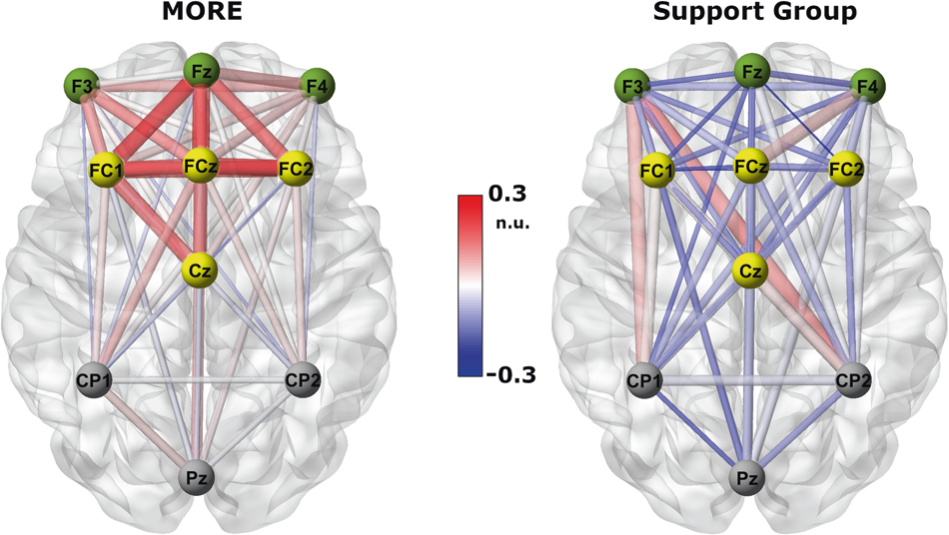
Theta coherence changes during a laboratory-based mindfulness meditation practice session before and after 8 weeks of mindfulness-oriented recovery enhancement (MORE) or a supportive group (SG) psychotherapy control condition. Red colors indicate positive coherence while blue colors indicate negative coherence. Saturation of color as well as line thickness represent the strength of coherence between nodes. ROIs are grouped via node color. This figure was created using BrainNet Viewer [55]. n.u. = normalized units.

The RM-ANOVA for theta spectral coherence changes revealed a significant treatment X time X ROI interaction effect, *F*(5,60) = 3.69, *p* = 0.003 (Fig. 2). This effect was driven by a significantly greater increase in FMT coherence for the MORE group relative to the SG, *F*(1,60) = 5.43, *p* = 0.023. In a sensitivity analysis controlling for opioid dose as well as recent benzodiazepine and THC use, treatment X time X ROI interactions for theta coherence remained statistically significant.

We then conducted two sets of additional sensitivity analyses by removing gross artifacts identified by visual inspection (1) before and (2) after processing with the PREP pipeline, and this did not alter the significance or directionality of the observed Group X Time interactions on spectral power or coherence.

### Effects of MORE on reducing opioid dose

In a linear mixed model controlling for pre-treatment differences in opioid dose, the treatment X time interaction was significant, *F*(1,110.27) = 5.50, *p* = 0.02, indicating that participants in MORE exhibited a greater decrease in opioid dose over time (estimated marginal mean MME at pre-treatment=94.6 ± 36.1 mg; estimated marginal mean MME at 4-month follow-up=79.72 ± 36.16 mg) than those in the SG (estimated marginal mean MME at pre-treatment=100.35 ± 40.54 mg; estimated marginal mean MME at 4-month follow-up=98.18 ± 40.58 mg).

### Association between changes in EEG spectral power and opioid dose

**Fig. 3:**
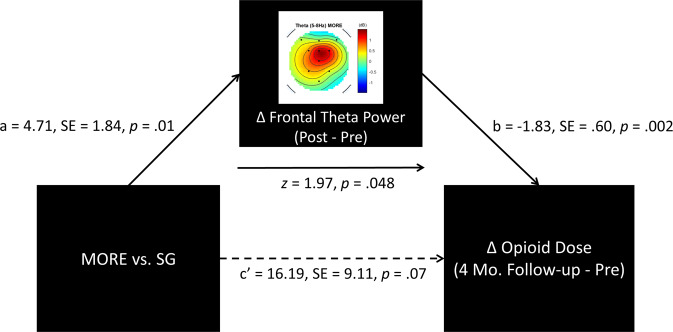
Path model testing frontal theta power as a mediator of reduced opioid dosing. Path model indicating that the effect of mindfulness-oriented recovery enhancement (MORE) versus a supportive group (SG) psychotherapy control condition on reducing opioid dose was statistically mediated by increasing frontal theta power mindfulness meditation.

Across the entire sample, increases in frontal theta power correlated with decreases in opioid dose by 4-month follow-up, *r* = −0.29, *p* = 0.049. Given this association, we conducted a path analysis, and found that significant ‘a’ and ‘b’ paths coupled with reduction in strength of the ‘c’ path in the path model suggested the presence of mediation (Fig. 3). Sobel test results indicated that the indirect effect of MORE on reducing opioid dose by increasing frontal theta power was statistically significant, *z* = 1.97, *p* = 0.048.

### Associations between changes in EEG spectral power and mindfulness-induced changes in self-referential processing

**Fig. 4:**
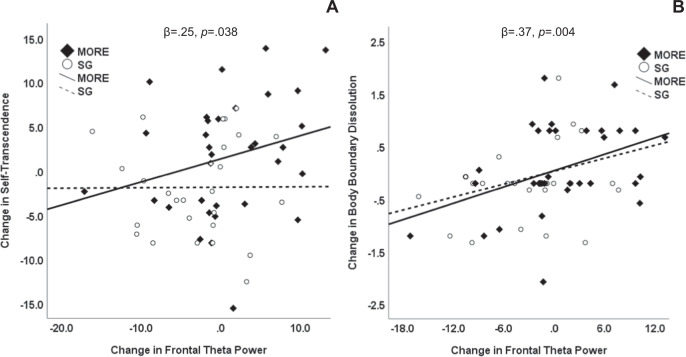
Associations between changes in frontal theta power and self-referential processing. Scatterplots depicting associations between treatment-related changes in frontal theta power and **a** self-transcendence (measured by the Nondual Awareness Dimensional Assessment) and **b** change in body boundaries (measured by the perceived body boundaries scale), among Veterans treated with mindfulness-oriented recovery enhancement (MORE) or a supportive group (SG) psychotherapy condition.

Across the entire sample, increases in frontal theta power predicted higher mindfulness-induced self-transcendence at post-treatment, controlling for pre-treatment levels, β = 0.25, *p* = 0.038. Inspection of within-group correlations (Fig. 4a) indicated that the association between changes in frontal theta and self-transcendence were stronger among participants in MORE than in the SG. Across the entire sample, increases in theta power across all electrode sites (*β* = 0.33, *p* = 0.01) predicted greater body boundary dissolution at post-treatment, controlling for pre-treatment levels; this association was most robust at frontal sites (*β* = 0.37, *p* = 0.004). Inspection of within-group correlations (Fig. 4b) indicated that the association between changes in frontal theta and body boundary dissolution were comparable between MORE and SG participants. Increases in frontal (*β* = 0.30, *p* = 0.02) but not central or parietal alpha also predicted greater body boundary dissolution at post-treatment.

### Association between changes in EEG spectral power and mindfulness practice duration

Within the MORE group, the number of minutes of daily mindfulness practice was positively associated with changes in FMT coherence (*r* = 0.35, *p* = 0.04), frontal theta (*r* = 0.33, *p* = 0.06), and central alpha power (*r* = 0.35, *p* = 0.04).

## Discussion

Mindfulness-based interventions are emerging as efficacious treatment options for an array of psychiatric disorders [42], and meta-analytic evidence supports their efficacy for reducing pain and opioid dosing [43]. While a considerable body of research demonstrates that mindfulness modulates neurophysiological oscillations in alpha and theta frequency ranges [15, 16], few studies have directly examined the effects of mindfulness training on EEG power in clinical samples. In this mechanistic study of Veterans with chronic pain receiving LTOT, we found that a novel mindfulness-based intervention, MORE, increased alpha and theta power across frontal, central, and parietal sites and FMT coherence during mindfulness meditation—oscillatory changes that were associated with mindfulness-induced changes in self-referential processing. Crucially, we found that MORE significantly reduced opioid dosing, and the effects of MORE on reducing opioid dose were statistically mediated by increases in frontal theta power during mindfulness meditation. Thus, increasing frontal theta power through mindfulness meditation may have important clinical consequences for pain patients on LTOT.

In humans with addictive behaviors, exogenous theta stimulation may be therapeutic. For example, among people with nicotine addiction, excitatory theta burst stimulation to the right inferior frontal gyrus of the PFC significantly increased inhibitory control [44]—a neurocognitive mechanism shown to predict decreased smoking relapse vulnerability [45]. In parallel, increased frontal theta and FMT power indicate heightened PFC activation [16, 20, 23], and meditation training increases mPFC and inferior frontal gyrus activation while reducing cigarette smoking [46]. The current study findings suggest that mindfulness meditation might produce endogenous theta stimulation in the PFC, thereby enhancing inhibitory control over opioid dose escalation. Future interventions combining exogenous (i.e., neurostimulation and/or neurofeedback technologies) and endogenous theta stimulation (i.e., mindfulness meditation) might produce an especially potent therapy for opioid misuse, OUD, and other substance use disorders.

Furthermore, prior human neuroimaging studies have suggested that sustained FMT power and coherence enhance myelination, measured via functional anisotropy, between the ACC and limbic structures, leading to greater anatomical connectivity after as few as four weeks of mindfulness training in healthy controls [23, 47, 48]. Supporting this notion, in preclinical mice models, theta stimulation increased the output of oligodendrocytes (cells responsible for myelin production) in the ACC while reducing anxiety [49, 50]. Thus, it is possible that the increases in theta power and coherence observed in the present study reflected the effects of prolonged meditation practice on brain plasticity. In that regard, we observed that Veterans who engaged in a longer total duration of mindfulness practice over the course of the study exhibited larger increases in FMT coherence and theta and alpha power during the laboratory-based meditation practice session. Given evidence that repeated practice of cognitive and behavioral skills [51] can induce changes in experience-dependent gene expression undergirding neuroplasticity [52], recurrent mindfulness practice might facilitate remodeling of neuroanatomical connections between top-down and bottom-up brain structures crucial to self-regulation of opioid use in the context of pain.

From a psychobiological perspective, deepening of the meditative state might be reflected in increased theta and alpha oscillations, which are thought to be most pronounced in long-term meditators [16, 18]. However, here in a sample of novices with substantial clinical vulnerabilities (i.e., chronic pain and opioid use), we observed significant increases in alpha and theta power and FMT coherence after 8 weeks of MORE that were indicative of the capacity to achieve deeper states of mindfulness. In support of this contention, increased meditation-induced alpha and theta oscillations were associated with marked alterations in self-referential processing during meditation – including ego dissolution and a blissful sense of oneness between self and world. These neurophenomenological correlates provide another hypothetical explanation for the reduction in opioid dose observed in the present study. The experience of self-transcendence occasioned by reduced self-referential processing during deep states of mindfulness might produce a potent experience of natural reward undergirded by increased functional connectivity among default mode, dorsal attentional, and salience networks [8]. In turn, enhancing natural reward via mindfulness practice is theorized to restructure reward processing by shifting valuation of drug-related rewards back to valuation of natural rewards, and thereby reducing addictive behavior [53]—a contention supported by recent neurophysiological data in chronic opioid users [31]. This possibility is especially intriguing, given the role of glutamatergic mPFC activity in both ego dissolution occasioned by psychedelics [54], reward, and the regulation of chronic opioid use [4]. Thus, Veterans who learned to self-generate internal reward via meditation may have been less compelled to consume opioids to obtain hedonic equilibrium, which may have accounted for the observed opioid sparing effects of MORE.

The current study had several limitations. First, a high density electrode montage would have allowed source localization for inferences about the brain networks involved. However, given the vulnerable nature of the study participants, we selected a limited set of electrodes to minimize participant burden. Second, because participants were instructed to take opioids as prescribed on the day of the experiment to prevent withdrawal-related cognitive and neurophysiological disturbances, the acute pharmacological effects of opioids may have influenced neurophysiological responses. That said, the observed effects of MORE on EEG power and coherence remained significant in sensitivity analyses controlling for opioid dose. Future studies could examine mindfulness-induced modulation of EEG oscillations in opioid-naïve chronic pain patients and in patients following stabilized medical tapering from LTOT. Also, the study had a modest sample size; future investigations should employ larger samples to examine associations between EEG parameters and a range of clinical outcomes. Finally, it is possible that decreases in opioid dosing might have driven some of the observed EEG changes, rather than the reverse; future studies could assess EEG at multiple follow-up points to further parse time-ordered relationships between changes in opioid dosing and neurophysiological responses. Furthermore, increases in EEG power and FMT coherence may simply be a marker of how skilled a practitioner is at meditating rather than the mechanism by which meditation leads to decreased opioid use. To rule out this alternative hypothesis, future studies could employ exogenous theta burst stimulation to facilitate and inhibit brain function in the absence of meditation and examine effects on opioid dosing.

In conclusion, following 8 weeks of treatment with MORE, Veterans receiving LTOT for chronic pain exhibited increased theta and alpha power, as well as enhanced FMT coherence, during a closed-eye mindfulness meditation that predicted decreases in opioid dose four months post-treatment. Given neural evidence of clinical target engagement, adequately powered, full-scale clinical trials are now needed to test the efficacy of MORE and other MBIs as opioid sparing interventions among people suffering from chronic pain.

## Funding and Disclosure

This work was supported by supported by W81XWH-16-1-0522 from the Department of Defense (PI: Garland) and R01DA042033 from the National Institute on Drug Abuse (PI: Garland). The content is solely the responsibility of the authors and does not necessarily represent the official views of the National Institutes of Health. The content is solely the responsibility of the author and does not necessarily represent the official views of the Department of Defense or National Institutes of Health. Eric Garland, PhD, LCSW is the Director of the Center on Mindfulness and Integrative Health Intervention Development. The Center provides Mindfulness-Oriented Recovery Enhancement (MORE), mindfulness-based therapy, and cognitive behavioral therapy in the context of research trials for no cost to research participants; however, Dr. Garland has received honoraria and payment for delivering seminars, lectures, and teaching engagements (related to training clinicians in mindfulness) sponsored by institutions of higher education, government agencies, academic teaching hospitals, and medical centers. Dr. Garland also receives royalties from the sale of books related to MORE. The remaining authors have nothing to disclose.
