# Correction: Endogenous theta stimulation during meditation predicts reduced opioid dosing following treatment with Mindfulness Oriented Recovery Enhancement

**DOI:** 10.1038/s41386-020-00925-z

**Published:** 2020-12-16

**Authors:** Justin Hudak, Adam W. Hanley, William R. Marchand, Yoshio Nakamura, Brandon Yabko, Eric L. Garland

**Affiliations:** 1 Center on Mindfulness and Integrative Health Intervention Development, University of Utah, Salt Lake City, UT USA; 2 College of Social Work, University of Utah, Salt Lake City, UT USA; 3 Veterans Health Care Administration VISN 19 Whole Health Flagship site located at the VA Salt Lake City Health Care System, 500 Foothill, Salt Lake City, UT 84148 USA; 4 Department of Psychiatry, University of Utah School of Medicine, 501 Chipeta Way, Salt Lake City, UT 84108 USA; 5 Department of Anesthesiology, Division of Pain Medicine, Pain Research Center, University of Utah School of Medicine, Salt Lake City, UT 84108 USA

**Keywords:** Human behaviour, Addiction

Correction to: *Neuropsychopharmacology* 10.1038/s41386-020-00831-4, published online 12 September 2020

This article was published electronically on the publisher’s internet portal on 12 September 2020 without open access. With the author(s)’ decision to opt for Open Choice, the copyright of the article changed on 20 November 2020 to © The Author(s) 2020 and the article is forthwith distributed under the terms of the Creative Commons Attribution 4.0 International License, which permits use, sharing, adaptation, distribution and reproduction in any medium or format, as long as you give appropriate credit to the original author(s) and the source, provide a link to the Creative Commons license, and indicate if changes were made. The images or other third party material in this article are included in the article’s Creative Commons license, unless indicated otherwise in a credit line to the material. If material is not included in the article’s Creative Commons license and your intended use is not permitted by statutory regulation or exceeds the permitted use, you will need to obtain permission directly from the copyright holder. To view a copy of this license, visit http://creativecommons.org/licenses/by/4.0/.

